# Mid-lateral cerebellar complex spikes encode multiple independent reward-related signals during reinforcement learning

**DOI:** 10.1038/s41467-021-26338-0

**Published:** 2021-11-09

**Authors:** Naveen Sendhilnathan, Anna Ipata, Michael E. Goldberg

**Affiliations:** 1 Doctoral Program in Neurobiology and Behavior, Columbia University, New York, NY USA; 2 Department of Neuroscience, Columbia University, New York, NY USA; 3 Mahoney Center for Brain and Behavior Research, Columbia University, New York, NY USA; 4 Zuckerman Mind, Brain, and Behavior Institute, Columbia University, New York, NY USA; 5 New York State Psychiatric Institute, New York, NY USA; 6 Kavli Institute for Brain Science, Columbia University, New York, NY USA; 7 Department of Neurology, Psychiatry, and Ophthalmology, Columbia University College of Physicians and Surgeons, New York, NY USA

**Keywords:** Learning and memory, Cerebellum, Reward

## Abstract

Although the cerebellum has been implicated in simple reward-based learning recently, the role of complex spikes (CS) and simple spikes (SS), their interaction and their relationship to complex reinforcement learning and decision making is still unclear. Here we show that in a context where a non-human primate learned to make novel visuomotor associations, classifying CS responses based on their SS properties revealed distinct cell-type specific encoding of the probability of failure after the stimulus onset and the non-human primate’s decision. In a different context, CS from the same cerebellar area also responded in a cell-type and learning independent manner to the stimulus that signaled the beginning of the trial. Both types of CS signals were independent of changes in any motor kinematics and were unlikely to instruct the concurrent SS activity through an error based mechanism, suggesting the presence of context dependent, flexible, multiple independent channels of neural encoding by CS and SS. This diversity in neural information encoding in the mid-lateral cerebellum, depending on the context and learning state, is well suited to promote exploration and acquisition of wide range of cognitive behaviors that entail flexible stimulus-action-reward relationships but not necessarily motor learning.

## Abstract (web summary)

The role of complex spikes in reinforcement learning is still unclear. Here, the authors show that complex spikes carry multiple context based, cell type specific and learning dependent signals that are independent of changes in any motor kinematics and unlikely to instruct the concurrent simple spike activity during reinforcement learning.

## Introduction

The cerebellum has been classically considered to be a center for supervised motor learning in the brain, where the predicted results of movement are compared with the animal’s actual performance, in order to correct the errors in the action that led to the mismatch^1–4^. The cerebellar cortex has been posited to achieve this via its two distinct types of inputs to its principle output cells, the Purkinje cells (P-cells). First, the mossy fibers, relayed through the parallel fibers of the granule cells, contain a number of sensory and efference copy signals, which are read out as high frequency simple spikes (SS)^5^. Second, the climbing fibers arising from the inferior olive (IO), which evoke complex spikes (CS), signal unexpected events or errors to facilitate learning^6^. The precisely timed relationship between the coincidence of CS and SS causes synaptic plasticity at the granule cell->P-cell synapse, thereby effecting learning. One such mechanism is long-term depression (LTD)^1,2,4,7^. This flow of information and circuitry explains many simple motor learning behaviors: connections that led to erroneous and undesirable behavior could be carefully pruned by the instructions provided by the CS.

However, motor learning and optimization do not always entail CS activity providing a teaching signal for SS responses^8–10^. Furthermore, recent evidence suggest that cerebellar activity is correlated with aspects of behavior that do not involve correcting the kinematics of movement: for example classical conditioning^11^, stimulus prediction^12,13^, and the magnitude of predicted reward^14,15^. The cerebellum’s role in these aspects of reward-related learning behavior cannot be readily explained by the present classical error-based learning models, nor do they necessarily entail CS activity affecting SS responses^14^ by an LTD mechanism. This is because, in reward-based learning, rather than pruning connections that led to erroneous behavior, the brain must strengthen connections that would lead to the preferred behavior^16^.

When the non-human primates learn to associate arbitrary visual symbols with hand movement choices, the SS encode a reinforcement error signal during learning, which gradually diminishes through learning, and disappears once the learning is completed^17^. This error signal, which could contribute significantly to reinforcement learning^18^, is encoded as the difference in SS activity between recent correct and wrong outcomes of P-cells^17^. However, (a) the role of concurrent CS activity, (b) the interaction between SS and CS, and (c) their relationship to complex reinforcement learning and decision making are all still unknown. Here, we show that while the SS carry a reinforcement learning signal, which has information about the outcome of the animal’s most recent decision, the concurrent CS do not carry such information nor do they instruct a change in SS’s activity. Instead, the CS encoded two different signals: first, a response to the beginning of the trial that may have predicted the possibility of reward given successful performance of the task, independent of both the state of reinforcement learning and the cell type. Second, a cell type and learning-state-specific learning response that occurred after two specific events: the symbol onset and the animal’s decision, describing the general probability of failure but not the actual outcome of the prior or current trial. Neither of these types of signals correlated with any changes in the motor kinematics.

These results show that although the mid-lateral cerebellum contributes to reinforcement learning^18^, the mechanism by which this learning occurs does not require CS-induced changes at the parallel fiber-P-cell synapse through an error-based mechanism. Rather, CS and SS form two independent channels of information, both encoding different aspects of reward-based learning depending on the context. Such differences in neural information encoding in the mid-lateral cerebellum and their complex interplay depending on the context and learning state may promote exploration and acquisition of wide range of cognitive behaviors that entail flexible stimulus–action–reward relationships.

## Results

**Fig. 1:**
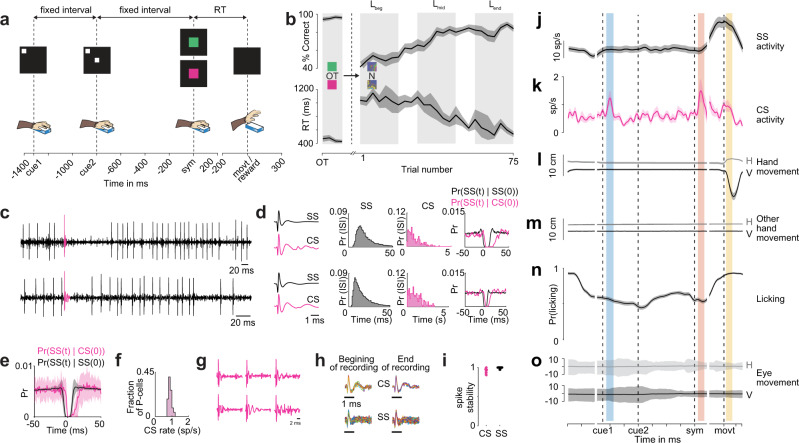
Experimental task, behavior, and cerebellar electrophysiology. **a** Schematic of a trial structure of the task. **b** Mean learning curve (top) and mean reaction time (bottom) from all sessions. *n* = 25 sessions. **c** Two representative raw neural signals from non-human primate B (top) and non-human primate S (bottom) highlighting the CS in pink. **d** Top panel: left: mean SS and CS waveforms from the cell shown in top panel of **c**. Middle: distribution of SS and CS interspike intervals (ISI) for the same cell. Right: conditional probabilities of spike timings. Bottom panel: same as the top panel but for cell shown bottom panel of **c**. **e** Average conditional probabilities of spike timings from all P-cells. *n* = 25 P-cells. **f** Population CS rate histogram. *n* = 25 P-cells. **g** Six representative CS waveforms from different P-cells showing the diversity in duration of CS and number of spikelet. **h** CS and SS waveforms from a representative recording at the beginning and end of recording. **i** Correlation of CS waveforms (left) and SS waveforms (right) between the beginning and the end of recording for each neuron used in this study. Correlations close to one indicates stability of recorded waveforms through time. *n* = 25 P-cells. **j** Mean spike density function of population SS responses in the OT condition. Blue shaded region indicates the cue epoch, orange, symbol epoch and yellow, reward epoch. *n* = 25 P-cells. **k** Mean spike density function of population CS responses in the OT condition. *n* = 25 P-cells. **l** Mean horizontal (gray) and vertical (black) positions of the responding hand. *n* = 25 sessions. **m** Mean horizontal (gray) and vertical (black) positions of the non-responding hand. *n* = 25 sessions. **n** Mean licking activity. *n* = 25 sessions. **o** Mean horizontal (gray) and vertical (black) eye positions. *n* = 25 sessions. Data shown as mean ± s.e.m. Source data are provided as a Source Data file.

Two non-human primates performed a two-alternative forced-choice discrimination task where, in each session, they associated one of two visual symbols with a left-hand movement and the other visual symbol with a right-hand movement^17^. They grabbed the two bars, each with one hand to initiate the trial. A small square (cue1) appeared on the top-left corner of the screen briefly (see Methods). After a fixed duration (523 ms), cue1 reappeared in the same position, along with another cue (cue2) at the center of the screen. Again, after a fixed duration (800 ms), one of the two symbols briefly appeared on the screen and they released the hand associated with that symbol, as soon as possible, with a well-learned stereotypic hand movement to earn a liquid reward (delivered 1 ms after correct movement onset) (Fig. 1a). The kinematics or the dynamics of hand movement were task irrelevant and only the choice of hands used to release the bars (associated with the symbols) merited reward. The animals usually performed ~30 trials of an overtrained (OT) association at the beginning of each session. Then, we presented them with two novel symbols that they learned to associate with specific choices (hand releases), through trial and error. They typically achieved criterion for learning (see Methods) in ~50–70 trials on an average through an adaptive learning mechanism (Fig. 1b). Their reaction time was high during early learning and decreased significantly through learning (Fig. 1b). The animals were free to move their eyes and thus occasionally made task-irrelevant eye movements.

Here we analyzed the CS activity P-cells recorded in Crus I and II of the non-human primate cerebellum whose SS activity we previously reported^17^ (see Methods). We identified P-cells by the presence of CS online (Fig. 1c), and offline by the (i) spike waveforms (Fig. 1d), (ii) the SS and CS interspike interval distribution (Fig. 1d), and (iii) a pause in SS after a CS (Fig. 1d, e)^19^. The CS fired at a very low firing rate, and in a minority of trials, consistent with prior reports^20,21^ (Fig. 1f), although they varied in number of spikelets and duration^21^ (Fig. 1g). We only analyzed activity from those cells (*n* = 25) with reliably detected CS that were stable throughout the entire recording (see Methods; Fig. 1h, i).

### P-cell response characteristics during the overtrained task

During the OT condition, the SS activity significantly changed from the baseline only during the hand movement (Fig. 1j). In contrast, there were significant changes in CS responses in three epochs: after the cue1 onset (cue1 epoch), after the symbol onset (symbol epoch), and after the animal’s decision (reward epoch) (Fig. 1k; see Methods). The majority of the cells responded in more than one epoch (Supplementary Table S1). The CS responses in any of the three epochs could not be explained by any obvious changes in motor kinematics, such as movement of the responding hand (Fig. 1l), the non-responding hand (Fig. 1m), licking (Fig. 1n), or eye movements (Fig. 1o).

The CS responded only in about 20% of trials in the cue1 epoch, 21% of trials in the symbol epoch, and in 19% of trials in the reward epoch (Supplementary Fig. S1a). Furthermore, we did not see any modulation in CS waveform duration among these three epochs (Supplementary Fig. S1b). The CS responses in the symbol epoch and during hand movement were not selective for the symbol (Supplementary Fig. S2a) or the choice of hand respectively (Supplementary Fig. S2b).

### CS activity after symbol onset was cell type specific and learning dependent

The mid-lateral cerebellar P-cell SS encode a reinforcement error signal when animals learn a new visuomotor association, by reporting the outcome of the most recent decision in short epochs called “delta epochs” in a manner entirely independent of the kinematics of the movement with which the animal made the response, or the various sensory events associated with reward delivery^17^. During learning, roughly half of the P-cells were selective for the wrong outcome (wP-cells; Supplementary Fig. S3a) and the remaining were selective for the correct outcome (cP-cells; Supplementary Fig. S3b) during these delta epochs^17^. The difference between the SS activities of the cP and wP-cells provides the error signal, which approaches zero as the animals learn the new association^17^.

We studied the learning-related changes in the CS activity after the symbol presentation in cP-cells (*n* = 14 cells) and wP-cells (*n* = 11 cells) separately. We analyzed the CS responses in 100 ms epoch (50–150 ms after symbol onset) in four different learning states (illustrated in Fig. 1b): last 20 trials of OT condition, the beginning of learning (L_beg_; the first 20 trials after the symbol switch), the middle of learning (L_mid_; the first 40–60 trials after the symbol switch), and at the end of learning (L_end_; 20 trials after the animal reached the criterion for learned; see Methods).

**Fig. 2:**
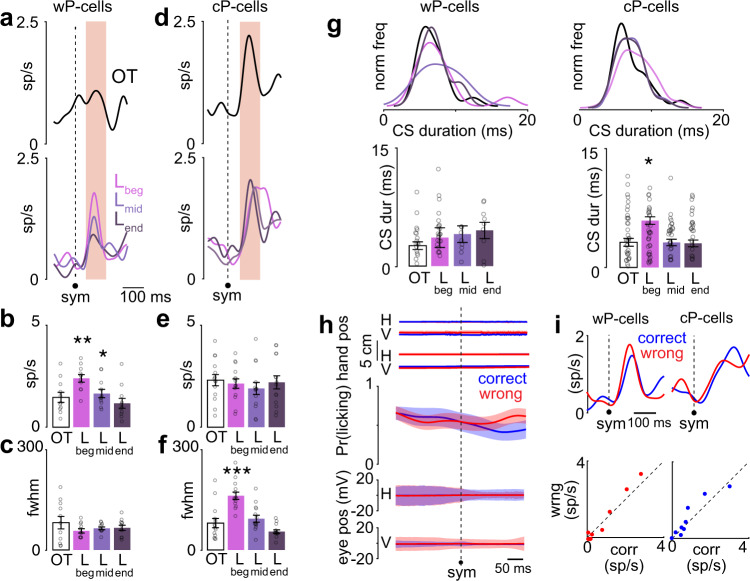
CS activity after symbol onset was cell type specific and learning dependent. **a** Top panel: spike density functions in the symbol epoch for wP-cells in the OT condition. Bottom panel: same for wP-cells in L_beg_, L_mid_, and L_end_. *n* = 11 wP-cells. **b** Peak firing rate of wP-cells in OT, L_beg_, L_mid_, and L_end_ conditions in the symbol epoch. **P < 0.01, *P < 0.05, two-tailed Wilcoxon signed rank test. *n* = 11 wP-cells. **c** Temporal dispersion of CS activity for wP-cells (estimated as the full width at half maximum firing rate) in OT, L_beg_, and L_end_ conditions in the symbol epoch. *n* = 11 wP-cells. **d** Same as **a** but for cP-cells. *n* = 14 cP-cells. **e** Same as **b** but for cP-cells. n = 14 cP-cells. **f** Same as **c** but for cP-cells. ***P < 0.001, two-tailed Wilcoxon signed rank test, *n* = 14 cP-cells. **g** Left: duration of CS waveforms in OT, L_beg,_ L_mid_, and L_end_ conditions for wP-cells (left, *n* = 11 wP-cells) and for cP-cells (right, *n* = 14 cP-cells). Histograms were normalized on the maximum frequency among all epochs. *P < 0.05, two-tailed Wilcoxon signed rank test. **h** From top to bottom: mean horizontal (H) and vertical (V) hand positions of the hand that was associated with the symbol presented, mean H and V hand positions of the hand that was not associated with the symbol presented, mean probability of licking, mean H and V eye positions for correct (blue) and wrong (red) trials. *n* = 25 sessions. **i** Top: CS activity during L_beg_ separated into correct (blue) and wrong (red) trials for wP-cells (left) and cP-cells (right). Bottom: scatter plot of peak neural activity during correct and wrong trials for individual wP-cells (left) and cP-cells (right). Data shown as mean ± s.e.m. Source data are provided as a Source Data file.

The CS peak firing rate of the wP-cells changed with learning: CS increased their firing rate during early learning from OT (OT-L_beg_: *P* < 0.01; two-tailed Wilcoxon signed rank test) and after learning, returned to an activity that was not different from OT (OT-L_end_: *P* = 0.24; two-tailed Wilcoxon signed rank test; Fig. 2a, b). However, the CS peak firing rate of cP-cells did not show any learning-related changes (*P* = 0.10, two-way Friedman test, 55 d.f. across all learning conditions that is, OT, L_beg_, L_mid_, and L_end_; Fig. 2d, e). Instead, the CS activity of cP-cells was more sustained or temporally dispersed (estimated as the full width at half maximum firing rate, fwhm) during learning, compared to the OT condition (OT-L_beg_: *P* < 0.001; two-tailed Wilcoxon signed rank test; Fig. 2d, f). After the animals learned the association between the symbols and the movements, the CS activity became temporally less dispersed (i.e., more temporally precise) as the symbols predicted a future reward more accurately (L_beg_ -L_end_: *P* < 0.001; two-tailed Wilcoxon signed rank test; Fig. 2d, f) and was no longer different from the OT condition (OT-L_end_: *P* = 0.22; two-tailed Wilcoxon signed rank test; Fig. 2d, f).

The duration of the CS waveform also differed during learning in a cell type-dependent way. Although the wP-cells did not show any learning-related changes in their CS waveform durations (*P* = 0.44, two-way Friedman test, 57 d.f. across all learning conditions; Fig. 2g), the CS waveform for cP-cells was longer at the beginning of learning compared to the OT condition (OT-L_beg_: *P* < 0.01, two-tailed Wilcoxon signed rank test; Fig. 2g) and decreased after learning, resembling the waveform in the OT condition (OT-L_end_: *P* = 0.06, two-tailed Wilcoxon signed rank test; Fig. 2g).

During learning, after the symbol onset, there were no changes in motor kinematics of the non-human primate (hand movement of the responding hand, the non-responding hand, licking, or the eye movement) and the motor behavior did not differ between correct and wrong trials (Fig. 2h). After the symbol onset, neither type of P-cells predicted the impeding decision’s outcome (Fig. 2i; wP-cell: *P* = 0.85, two-tailed Wilcoxon signed rank test, and cP-cell: *P* = 0.88, two-tailed Wilcoxon signed rank test).

### CS activity after the non-human primate’s decision was also cell type specific and learning dependent

**Fig. 3:**
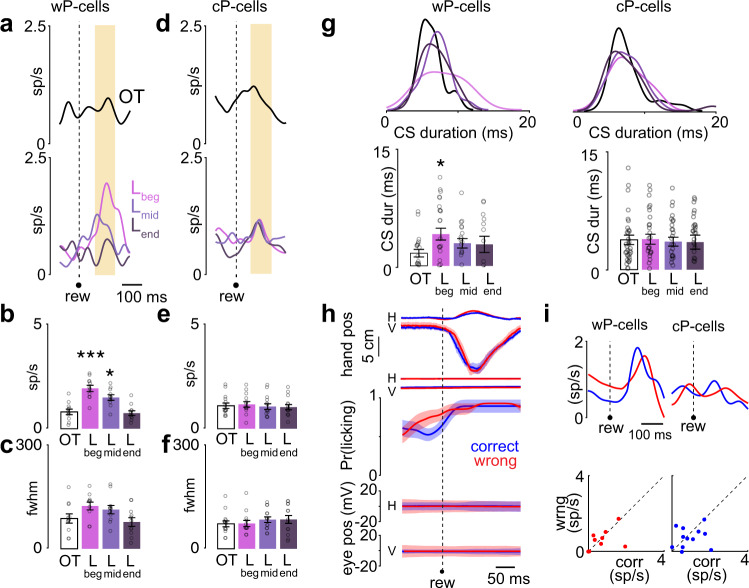
Only wP-cells encoded learning-dependent signal after the animal’s decision. **a** Top panel: CS spike density functions in the reward epoch for wP-cells in the OT condition. Bottom panel: same for wP-cells in L_beg_, L_mid_, and L_end_. *n* = 11 wP-cells. **b** Peak firing rate of wP-cells in OT, L_beg_, L_mid_, and L_end_ conditions in the reward epoch. ***P < 0.001, *P < 0.05, two-tailed Wilcoxon signed rank test. *n* = 11 wP-cells. **c** Temporal dispersion of CS activity for cP-cells (estimated as the full width at half maximum firing rate) in OT, L_beg_, L_mid_, and L_end_ conditions in the reward epoch. *n* = 11 wP-cells. **d** Same as **a** but for cP-cells. *n* = 14 cP-cells. **e** Same as **b** but for cP-cells. *n* = 14 cP-cells. **f** Same as **c** but for cP-cells. *n* = 14 cP-cells. **g** Left: duration of CS waveforms in OT, L_beg_, L_mid_, and L_end_ conditions for wP-cells (left, *n* = 11 wP-cells) and for cP-cells (right, *n* = 14 cP-cells). Histograms were normalized on the maximum frequency among all epochs. *P < 0.05, two-tailed Wilcoxon signed rank test. **h** From top to bottom: mean horizontal (H) and vertical (V) hand positions of the hand that was associated with the symbol presented, mean H and V hand positions of the hand that was not associated with the symbol presented, mean probability of licking, mean H and V eye positions for correct (blue) and wrong (red) trials. *n* = 25 sessions. **i** Top: CS activity during L_beg_ for correct (blue) and wrong (red) trials for wP-cells (left) and cP-cells (right). Bottom: scatter plot of peak neural activity for correct and wrong trials for individual wP-cells (left) and cP-cells (right). Data shown as mean ± s.e.m. Source data are provided as a Source Data file.

CS activity in the reward epoch was also cell type specific. Here, the firing rate of wP-cells significantly increased at the beginning of learning from OT (OT-L_beg_: *P* < 0.01, two-tailed Wilcoxon signed rank, Fig. 3a, b) and decreased to a lower activity in the mid learning and finally decreasing even further, comparable to the activity in the OT condition after the animals learned the task (OT-L_end_: *P* = 0.90, two-tailed Wilcoxon signed rank test, Fig. 3a, b). There were no learning-related changes in the temporal dispersion (*P* = 0.61 two-way Friedman test, 42 d.f. across all learning conditions, Fig. 3a, c). However, across learning, the cP-cells did not show any significant learning-related changes either in their peak firing rate (*P* = 0.33, two-way Friedman test, 54 d.f. across all learning conditions, Fig. 3d, e) or the temporal dispersion of activity (*P* = 0.36, two-way Friedman test, 52 d.f. across all learning conditions; Fig. 3d, f).

Consistent with learning-related changes in peak firing rate for wP-cells, the duration of CS was longer during the beginning of learning compared to the OT condition (OT-L_beg_: *P* < 0.05 two-tailed Wilcoxon signed rank test, Fig. 3g) and the duration decreased after learning and was comparable to OT (OT-L_end_; *P* = 0.16, two-tailed Wilcoxon signed rank test; Fig. 3g). The CS waveform duration for cP-cells did not change in this epoch during learning (*P* = 0.21, two-way Friedman test, 55 d.f. across all learning conditions; Fig. 3g).

Finally, during learning, after the non-human primate’s decision, there were no changes in motor kinematics of the non-human primate (hand movement of the responding hand, the eye movement, non-responding hand, licking) between correct and wrong trials (Fig. 3h). Neither type of P-cells reported the recent decision’s outcome (Fig. 3i; cP-cell: *P* = 0.31 and wP-cell: *P* = 0.88 two-tailed Wilcoxon signed rank test), contrary to prior reports^13^. They did not predict the next trial’s outcome either (Supplementary Fig. S4).

### CS responded to the stimulus that signaled the beginning of the trial

**Fig. 4:**
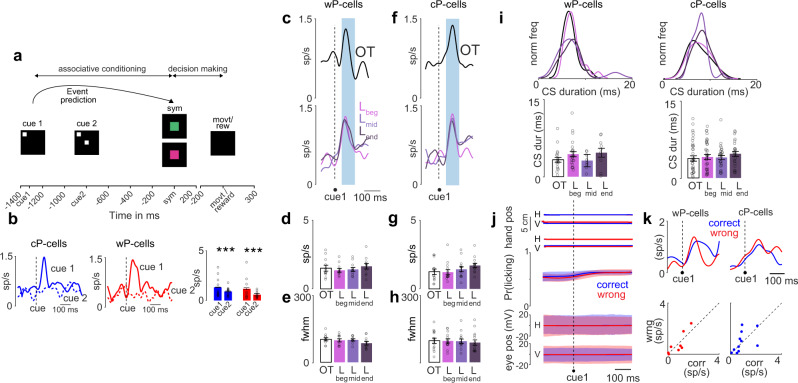
CS responded to the stimulus that signaled the beginning of the trial but not to a second, temporally paired stimulus. **a** Trial structure with a schematic of event prediction. **b** Left: CS activity for cue1 and cue2 for cP-cells (left; blue) and wP-cells (right; red). Right: quantitation from left panel. ***P < 0.001, two-tailed Wilcoxon ranksum test. **c** Top panel: CS spike density functions in cue1 epoch for wP-cells in the OT condition. Bottom panel: same for wP-cells in L_beg_, L_mid_, and L_end_. *n* = 11 wP-cells. **d** Peak firing rate of wP-cells in OT, L_beg_, L_mid_, and L_end_ conditions for cue1 epoch. *n* = 11 wP-cells. **e** Temporal dispersion of CS activity for wP-cells (estimated as the full width at half maximum firing rate) in OT, L_beg_, L_mid_, and L_end_ conditions in the reward epoch. *n* = 11 wP-cells. **f** Same as **c** but for cP-cells. *n* = 14 cP-cells. **g** Same as **d** but for cP-cells. *n* = 14 cP-cells. **h** Same as **e** but for cP-cells. *n* = 14 cP-cells. **i** Left: duration of CS waveforms in OT, L_beg_, L_mid_, and L_end_ conditions for wP-cells (left, *n* = 11 wP-cells) and for cP-cells (right, *n* = 14 cP-cells). Histograms were normalized on the maximum frequency among all epochs. **j** From top to bottom: mean horizontal (H) and vertical (V) hand positions of the hand that was associated with the symbol presented, mean H and V hand positions of the hand that was not associated with the symbol presented, mean probability of licking, mean H and V eye position for correct (blue) and wrong (red) trials. *n* = 25 sessions. **k** Top: CS activity during L_beg_ for correct (blue) and wrong (red) trials for wP-cells (left) and cP-cells (right). Bottom: scatter plot of peak neural activity for correct and wrong trials for individual wP-cells (left) and cP-cells (right). Data shown as mean ± s.e.m. Source data are provided as a Source Data file.

On every trial, before we presented the symbols that instructed the hand movements, we presented two additional cues: cue1 and cue2 with a fixed interval of 523 ms between them (see Methods; Fig. 4a). Both types of P-cells only fired for cue1 but not for cue2. That is, for both types of P-cells, CS activity in response to cue1 was significantly higher than the baseline (cP-cells: *P* < 0.001; wP-cells: *P* < 0.001 two-tailed Wilcoxon signed rank test; Fig. 4b) and was significantly higher than that for cue2 (cP-cells: *P* < 0.001, wP-cells: *P* < 0.001, two-tailed Wilcoxon signed rank test; Fig. 4b), which was not different from the baseline value (cP-cells: *P* = 0.36; wP-cells: *P* = 0.54 two-tailed Wilcoxon signed rank test; Fig. 4b).

For both types of P-cells, there was no learning-related modulation in either the peak activity (wP-cells: *P* = 0.49, two-way Friedman test, 43 d.f.; Fig. 4c, d; cP-cells: *P* = 0.44, two-way Friedman test, 54 d.f. across all learning conditions; Fig. 4f, g) or temporal dispersion of activity (wP-cells: *P* = 0.18, two-way Friedman test, 40 d.f. across all learning conditions; Fig. 4c, e; cP-cells: *P* = 0.62, two-way Friedman test, 55 d.f. across all learning conditions; Fig. 4f, h). There were no changes in CS waveform duration between the two groups or through learning (wP-cells: *P* = 0.96, two-way Friedman test, 60 d.f., cP-cells: *P* = 0.07, two-way Friedman test, 124 d.f. across all learning conditions, Fig. 4i).

During this epoch, there were no changes in motor kinematics of the non-human primate (hand movement of the responding hand, the eye movement, non-responding hand, or licking). And, the motor behavior did not differ between correct and wrong trials for any of the effectors (Fig. 4j). The CS activity in this epoch did not encode prior decision’s outcome (wP-cell: *P* = 0.36, two-tailed Wilcoxon signed rank test, cP-cell: *P* = 0.92, two-tailed Wilcoxon signed rank test, Fig. 4k).

### CS activity was unrelated to SS activity or behavior during learning of novel visuomotor associations

Finally, we investigated whether the CS activity related to the SS activity and the behavior during learning. In motor learning, CS acts as a teaching signal, instructing the SS output and the motor behavior through an error-based supervised learning framework^3^. However, we have several lines of evidence suggesting CS activity does not affect SS activity during learning of novel visuomotor associations

**Fig. 5:**
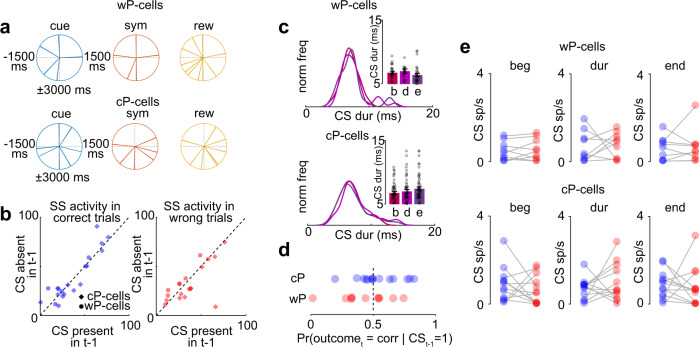
SS and CS formed independent channels of neural encoding during reinforcement learning. **a** Polar plots (of the entire trial period) of time of significant CS modulation during learning relative to the time of cue onset (left), symbol onset (middle) and reward onset (right) for each cell for all wP-cells (top) and cP-cells (bottom). Each line on the plot represents time of significant modulation of CS (in ms) relative to the respective event signifying the temporal relationship between delta epoch and CS activity. **b** Left: SS activity for wP-cells in correct (left) and wrong (right) trials with CS present in the previous trial (abscissa) vs CS absent in the previous trial (ordinate). Right: same as left; but for cP-cells. **c** Duration of CS waveforms at the beginning (b), during (m), and end (e) of delta epoch for wP-cells (left) and cP-cells (right). Histograms were normalized to the maximum frequency among all epochs. **d** Probability that a trial (t) during learning would be correct, given there was a CS in the previous trial (t – 1) in cP-cells (left) and wP-cells (right). **e** Peak CS activity for correct (blue) and wrong (red) trials during learning at the beginning (left), during (middle), or the end (right) of delta epoch for cP-cells (top) and wP-cells (bottom). Data shown as mean ± s.e.m. Source data are provided as a Source Data file.

First, the time of delta epoch was not temporally related to the time of CS activity in cue, symbol, or reward epoch for either type of P-cells (Fig. 5a; wP-cells: cue: *P* = 0.72, symbol: *P* = 0.42, reward: *P* = 0.59; cP-cells: cue: *P* = 0.43, symbol: *P* = 0.79, reward: *P* = 0.13 circular Rayleigh *z* test, see Supplementary Fig. S5a–c for single cell examples). Furthermore, 2/25 P-cells with delta epochs did not show any significant modulation in CS during any of the three times at which we found significant responses in the majority of P-cells (Supplementary Fig. S5d). This indicates that the time of delta epoch is unrelated to the time of CS responses during learning^17^ suggesting a causal dissociation between the two. That is, the CS activity did not cause the delta epoch during learning.

Second, during certain types of motor learning, for instance, smooth pursuit learning, CS activity has a profound effect on SS activity in on the next trial^20^. When non-human primates learn to predict a smooth pursuit direction change, the presence of a CS in the prior trial is associated with a decrease of SS activity in the current trial, which occurs 175–50 ms before the time at which the CS occurred in the prior trial, as if the presence of the CS depressed the response of the P-cell to the parallel fiber activity that had occurred during learning^20^. However, in our reinforcement learning task, if CS were present in the previous trial during learning, the SS activity in the next trial 175–50 ms before the CS was not different from the SS activity in the same epoch for which CS was absent on the previous trial. This was true both across trial type and cell type (Fig. 5b; correct trials: cP-cells: *P* = 0.89, wP-cells: *P* = 0.80, two-tailed Wilcoxon ranksum test, Pearson *r* = 0.91, *P* < 0.001; wrong trials: cP-cells: *P* = 0.51, wP-cells: *P* = 0.65, two-tailed Wilcoxon ranksum test, Pearson *r* = 0.69, *P* < 0.001).

Third, also in smooth pursuit learning, the duration of CS is longer during the instruction epoch compared to the fixation epoch (a task irrelevant epoch)^21^. In contrast, in our task, we found no changes in CS waveform duration (Fig. 5c) at the beginning, during, or end of delta epoch for either type of cells during learning.

Although CS activity is frequently correlated with some aspect of the non-human primate’s behavior, we have two lines of evidence that this is not the case in reward-based visuomotor association learning. First, the CS activity in the prior trial could affect the behavioral performance of the non-human primate in the next trial during motor learning. For example, during smooth pursuit learning, the presence of a CS in a given trial was associated with a change of pursuit velocity in the next trial^20,21^. Similarly, during a saccade adaptation task, the CS encoded the error in saccade amplitude and direction that allowed for correction of that error in the text trial, improving the behavioral performance. However, in our reinforcement learning task, if CS were present in the previous trial during learning, the probability that the next trial would be correct was not significantly higher than chance level (cP-cells: *P* = 0.42, wP-cells: *P* = 0.33, one sample *t*-test; Fig. 5d). This means that CS responses did not affect behavior through an error-based learning mechanism.

Second, the CS had no information about the outcome of the prior trial during learning, even at a time in the trial when the SS reported the outcome of the prior trial^17^. The CS activity at the beginning, during, or end of delta epoch during learning did not carry information about the prior trial outcome (Fig. 5e start of the delta epoch: cP-cells: *P* = 0.29, wP-cells: *P* = 0.81, two-tailed Wilcoxon signed rank test; middle of delta epoch: cP-cells: *P* = 0.75, wP-cells: *P* = 0.80, two-tailed Wilcoxon signed rank test; end of delta epoch cP-cells: *P* = 0.23, wP-cells: *P* = 0.75, two-tailed Wilcoxon signed rank test).

All these provide strong converging evidence that CS were unlikely to instruct a change in SS activity through the classical error-based learning framework^14,22^. This furthermore suggests that the CS neural activity is entirely unrelated to SS activity.

## Discussion

A comprehensive role for the cerebellum in reinforcement learning is not well understood. Several recent studies show cerebellar activity correlated with reward-based paradigms^12–15,17^. However, all these reinforcement learning-based studies have focused primarily on only one aspect of neural encoding in the cerebellum (either SS or CS). In this study, we show that when a non-human primate learns a new visuomotor association (Fig. 1), classifying CS responses based on their SS properties (depending on whether the SS preferentially encoded success on the prior trial, cP-cells, or failure, wP-cells)^17^ revealed distinct cell type-specific encoding of the probability of failure after the symbol onset (Fig. 2) and the animal’s decision (Fig. 3), but not the decision’s outcome (which is encoded by SS). CS from both cell types, from the same cerebellar area also responded to the symbol that signaled the beginning of the trial (Fig. 4). Importantly, all these CS signals were independent of changes in any motor kinematics (Figs. 2–4). The CS did not instruct changes in concurrent SS activity during reinforcement learning (Fig. 5), nor was CS activity related to the outcome of the prior or current trial.

### Multiple channels of information encoding in the cerebellum during reinforcement learning

Unlike studies of motor learning^20,23^ and in contrast to the classic Marr–Albus model of the cerebellum, we did not find any relationship between the learning properties of CS activity and that of SS activity. One might have expected that a CS signal could have served as a teaching signal for the delta epoch of SS during learning if the classical error correcting framework were to apply to non-motor learning^3^. This was not at all the case (Fig. 5). There are several reasons why CS signals are unlikely to play the role of a teaching signal in our experiment. First, at the symbol switch between the OT and learning conditions, the SS suddenly express large differences in activity in the delta epoch (~30 sp/s). It is unlikely that this difference in the SS rate could have been caused solely by synaptic depression elicited by CS that has only been shown to cause a maximum of 8–10 sp/s changes in SS activity (with the longest CS waveforms)^20,21^. In addition, if the CS were causing the delta epochs, we should have seen a tight temporal relationship between the two, but we did not. It may be that CS only provide error signals during certain types of motor learning, and not for other types of learning. For example, the CS in the flocculus signal both the expected amount of reward and the motor properties^14^.

During our reinforcement learning task, SS encode the magnitude of the reinforcement learning error, reporting the result of the most recent decision, while CS encode the probability of failure without having information about the result of the most recent decision. Both these signals disappear with learning (Figs. 2 and 3). This is in contrast with the recent reports in mice where the CS activity persists after learning, either reporting the trial outcome^13,15^ or predicting the reward^12^. The role of concurrent SS in these studies is unclear. Furthermore, in our task, the SS and CS signals form two distinct channels of neural information encoding during reinforcement learning as they do not seem to interact at the level of the cerebellar cortex (Fig. 5). However, they could impact downstream processing at the level of deep cerebellar nuclear (DCN) neurons (Supplementary Fig. S6).

Apart from the reward-based, learning-dependent, and cell type-dependent signals encoded by CS after symbol and the animal’s decision, the CS also encoded a learning- and cell type-invariant response to the cue1 that signaled the beginning of the trial that was also the first of a series of temporally paired stimuli (Fig. 4). Cue1 occurred at the beginning of the trial. After its presentation, the animal’s prediction that it would get a chance to earn a reward would change. However, after the presentation of cue2, the animal does not update its prediction since cue2 occurs after a fixed interval after cue1. Keeping with this, both types of P-cells only fired for cue1 but not for cue2. Because cue1 occurred at different times after correct or wrong trials (due to an additional timeout of 2200 ms after wrong trials, see Methods), it could have not been a late response to the termination of the hand movement in the prior trial^24^. The response was unlikely to be just a visual response to cue1: the same stimulus (as cue1) reappeared along with cue2, but the P-cells did not respond to it. Every cell that responded to cue1 also responded to the symbol and/or after the animal’s decision. The stimulus evoking the cue1 response appeared after the symbol but not after the decision, which shows that the stimulus per se was not necessary for the response. Because of the fixed timing between cue1 and the symbol appearance, it is possible that this was a learned response to a stimulus, which was similar to the conditioned stimulus of a classical Pavlovian association, in this case the appearance of the symbols. This is consistent with a temporal difference error signal^11^, although the signal was not linked to the presence of reward, but rather to the possibility of performing a task to earn a reward. Since we performed electrophysiological recordings months after training both non-human primates with repeated presentation of temporally paired stimuli, we could not confirm if both the cues originally evoked a CS response that migrated eventually to cue1. Nevertheless, since the appearance of cue1 always preceded the symbols (that instructed the hand movement), it could also serve as an alerting response preparing the animal for the trial.

Together, these results show that individual CS in the same cerebellar area are flexible in that they can encode very different non-motor signals, depending on the context—a reinforcement learning-dependent and cell type-dependent signal when the animal learns to make a decision, and a reinforcement learning-independent and cell type-independent response to the stimulus that signaled the beginning of the trial, consistent with a temporal difference error during classical conditioning. This mixed selectivity suggests new and general roles for CS signals that are disparate from classical error-based supervised learning.

### A cerebellar circuit that contributes to reinforcement learning

The reinforcement learning signal encoded by the SS could be a transformation of the reward signals provided by the granule cells^25^, which in turn receive convergent reward and sensory input from diverse brain areas. However, if the CS also carry reward-related information, where could this information come from? One such key source of input to the IO is the meso-diencephalic junction (MDJ)^26^, a midbrain region composed of multiple nuclei, some of which integrate DCN output and project to either downstream neurons in the IO^27^. The MDJ also integrates descending input from cortical pyramidal tract neurons^28^, thus allowing the IO to represent higher order cortical computations. This is a good candidate to transmit the types of reward-related information.

While CS activity in cP-cells showed both activity related to the probability of failure after both the symbol onset and decision, CS activity in wP-cells only showed the latter (Figs. 2 and 3). The waveform duration of CS also mirrored these changes. If different types of P-cells (cP-cells and wP-cells) projected to different types of DCN cells, and this segregation were maintained in the projection from the DCN to the IO, the IO neurons could maintain this functional difference as well. Therefore, just like there are cP-cells and wP-cells, we suggest that there may be cIO-cells and wIO-cells that project to these respective P-cell populations (Supplementary Fig. S6 shows schematics of the circuits by which P-cells with the two different types of CS could contribute to visuomotor association learning). However, unlike SS, the climbing fiber activity did not carry information about the most recent decision during learning. Extracellular recording in the non-human primates cannot provide information about functional or molecular segregation of P-cells. This is unlike the mouse, where functional differences in P-cells could be reflected in molecular expression of different proteins (Adolase or antigen, Zebrin)^29^ or differences in anatomical location (microzones)^13^. However, the neural basis of this functional differences in IO cells is yet unknown. Interestingly, although both these cell types responded to the stimulus that signaled the beginning of the trial in the same way in both the OT and learning contexts, they encoded different information during learning, suggesting that the information about the trial-beginning stimulus could be projected onto both cell types from an upstream to the IO.

Both the climbing fiber and ~50 P-cells^30^ project to a single DCN neuron. The two information channels (SS and CS) carrying different information (as discussed above) could sculpt the information encoded in the DCN (Supplementary Fig. S6). The DCN is connected to the striatum^31^ and the PFC^32^ through the thalamus and is monosynaptically connected to the ventral tegmental area (VTA)^33^. Optogenetic stimulation of the DCN reliably evokes postsynaptic responses in both GABAergic as well as dopaminergic VTA neurons, contributing to reward-related behavior and social behavior^34^. Suppressing this connection is sufficient to abolish social behavior in mice^34^. VTA dopaminergic neurons have two key downstream targets: the ventral striatum^35^ and the prefrontal cortex^36^ both of which have been shown to be critically involved in reward processing^37,38^.

Although it is clear from our results that the CS do not inform the SS about the results of the prior trial, other cerebellar structures might. The SS synapse, affected by the CS in motor learning, is not the only modifiable synapse in the cerebellum^39^. For example, the calcium responses of molecular layer interneurons become selective for the rewarded odorant as mice learn which of a pair of odorants is associated with a reward, and which with a punishment (a brief timeout) and an optogenetic inactivation of these cells slows the learning process^40^.

The question then arises whether the different signals encoded by the CS, at the beginning of the trial and the probability of failure, which have no relationship to trial-by-trial error or reward, could also contribute to the process of visuomotor association learning. One mechanism by which they could is to provide a parallel motivational signal through the cerebellar projections to the dopaminergic system via the DCN. The DCN neurons project to several dopaminergic areas, including the VTA^34^ and the substantia nigra pars compacta^41^. Dopamine neurons are not exclusively related to reward. Different dopamine neurons respond to alerting and motivating signals as well as reward^42^. The CS responses that we have discovered could, via the direct projection of the climbing fibers to the DCN, excite the midbrain dopamine system, providing a cerebellar contribution to behavior entirely independent from associative learning. A lack of this signal to the basal ganglia could contribute to the learning deficit caused by mid-lateral cerebellar inactivation. Our results suggest that the SS and CS in the cerebellum have signals that could be useful for two different networks in the brain, a traditional error signal in the SS that project to the sensorimotor network, and, possibly, a motivational or arousing signal from the CS, which projects to the dopaminergic system. This synergy between the sensorimotor and motivational contributions of cerebellar processes may provide the flexibility necessary for sophisticated cognitive functions.

## Methods

### Experimental model and subject details

We performed all experiments on two adult male non-human primates (*Macaca mulatta*), B (age: 14 years) and S (age: 7 years), weighing 10–11 kg each, for the experiments. All experimental protocols were approved by the Animal Care and Use Committees at Columbia University and the New York State Psychiatric Institute, and complied with the guidelines established by the Public Health Service Guide for the Care and Use of Laboratory Animals.

### Method details

#### Behavioral task

We used the NIH REX-VEX system for behavioral control. The non-human primates sat inside a dimly lit recording booth, with its head firmly fixed, in front of a back-projection screen upon which visual images were projected.

The two-alternative forced-choice discrimination task began with the non-human primates grasping two bar-manipulanda, one with each hand, after which two cues (white square) appeared sequentially. The first one was briefly flashed on the top-left corner of the screen to signal a photocell that there was a programming change in the VEX display system. This square appeared at every subsequent change in the video display. The computer began to monitor whether the non-human primates had pressed the bars 20 ms after this cue. On 97% of the trials, the non-human primates had pressed both bars during the inter-trial interval (ITI) and on those after a fixed interval of 525 ms, the second one was flashed at the center of the screen for 800 ms. On the remaining 3% of the trials, the non-human primates waited until after cue1 to press the bar, so there was a variable time between the two cues. Then one of a pair of fractal symbols, that the non-human primate had never seen before, appeared briefly for 100 ms, at the center of gaze. There was no jitter in time between the time of cue onset and the time of symbol onset. One symbol signaled the non-human primate to release the left bar and the other to release the right bar. We rewarded the non-human primates with a drop of liquid juice reward for releasing the hand associated with that symbol as soon as possible. From the initiation of the hand movement, there was an 800 ms delay (ITI) until the next trial started. On wrong trials, we increased this ITI from 800 ms to (800 ms ITI + 2200 ms timeout) 3000 ms, to increase the non-human primates’ motivation to perform the task. The non-human primates were free to move their eyes and make any hand movement as long as they released the correct bar associated with the presented symbol. Although this was the case, non-human primates made very stereotypic hand movements that did not change across trials.

In the OT condition, the non-human primates were repeatedly presented with the same familiar pair of symbols for which the non-human primates have learned the associations over 4–6 months. In the novel condition, the non-human primates were presented with a different pair of novel symbols that they have never seen before. They learned the association between these novel symbols and left- or right-hand release through trial and error. On every recording session, we started with the OT condition and after ~30 trials, switched to the learning condition.

A correct trial was defined as the trial in which the non-human primate released only the one correct hand associated with the symbol. The non-human primates received reward only for correct trials. We defined a wrong trial as the trial in which the non-human primate released the hand not associated with the symbol. Trials where the non-human primates released both hands anytime during the trial, or released the hand(s) before the symbol onset or released the hand(s) after 2800 ms from symbol onset were considered abort trials and were neither rewarded nor analyzed.

We constructed the learning curve for every session by calculating the percent correct trials in a sliding window of 10 trials shifted by 5 trials. If the non-human primates reached >90% correct through the above method and remained above 80% for at least the next 20 trials, the associations were considered “learned.”

#### Single unit recording

Here, we analyzed CS and SS activity from a previous study^17^. Briefly, we used two recording cylinders, on the left hemisphere of each non-human primate. We introduced glass-coated tungsten electrodes with an impedance of 0.8-1.2 MOhms (FHC) into the left mid-lateral cerebellum of non-human primates every day that we recorded using a Hitachi microdrive. We passed the raw electrode signal through a FHC Neurocraft head stage, and amplifier, and filtered through a Krohn-Hite filter (bandpass: lowpass 300 Hz to highpass 10 kHz Butterworth), then through a Micro 1401 system, CED electronics. We used the NEI REX-VEX system coupled with Spike2 (CED electronics) for event and neural data acquisition. We verified all recordings offline to ensure that we had isolated P-cells and that the spike waveforms had not changed throughout the course of each experiment. To do this, we correlated the spikes from the beginning and the end of a recording session and used only those sessions that had at least a correlation of 0.85 (Fig. 1h, i). The CS of 25 cells satisfied this criterion.

#### Hand tracking

We painted a spot on the non-human primates’ right hand with a UV-blacklight reactive paint (Neon Glow Blacklight Body Paint) prior to every session. We used a 5 W DC converted UV blacklight illuminator to shine light on the spot. Then we used a high speed (250 fps) camera (Edmund Optics), mechanically fixed to the primate chair, to capture a video sequence of the hand movement while the non-human primates performed the tasks. We used the track mate Image J^43,44^ and custom written software in MATLAB to semi-manually track the fluorescent paint spot painted on the non-human primate’s hand.

#### Licking

We recorded licking at a sampling rate of 1000 Hz using a capacitive touch sensor coupled to the metal water spout that delivered liquid water reward near the non-human primate’s mouth. Raw binary lick traces were used to generate instantaneous lick rate by trial averaging and smoothing it with a Gaussian kernel of sigma = 20.

#### Eye movements

We tracked the non-human primate’s left eye positions at 240 Hz sampling rate with an infrared pupil tracker (ISCAN, Woburn, MA USA) interfaced with Spike2 (CED electronics) where it was upsampled to 1000 Hz and synced with the event markers from NEI REX-VEX system.

### Quantification and statistical analysis

#### Quantitation of CS activity

To study the event related CS activity, for each cell, we first aligned the CS responses to cue1, cue2, symbol, and reward onset. Then, for each condition, we binned the CS responses in 1 ms bins and convolved the resulting function with a Gaussian kernel of sigma = 20 ms to obtain spike density functions, for each cell. Then, we quantified the firing rate and the temporal dispersion (estimated as the full width at half maximum firing rate, fwhm) in a 100 ms window (50–150 ms after respective event onset) for each condition, and averaged across single cell results to provide our final estimates. We confirmed the independence of these two measures through a lack of significant correlation.

#### Epochs of significant CS activity

We estimated the epochs where the CS had significant activity by performing a two-tailed *t*-test between the population CS activity (across all cells and all trials) in every 100 ms bins and a baseline activity (–100 to 0 ms aligned to cue2 onset). Then, we corrected for multiple comparisons using the Benjamini and Hochberg/Yekutieli false discovery rate method. Through this method, we found three epochs with significant CS activity: after cue1, symbol, and reward epochs (Supplementary Fig. S1). However, to be consistent in our analysis, we only analyzed data in 100 ms bins in all three epochs. Therefore, we analyzed the CS responses 50–150 ms after symbol onset, reward onset, and cue1 onset. Furthermore, we analyzed the data from a condition of a cell only if it had at least one CS across trials in that condition’s interval.

#### Measurements of CS morphology

The validity of the data presented in Figs. 2g, 3g, 4g, and 5c depends on the accuracy of our CS duration measurements. One of the authors manually made all these measurements while being blind to the type of cell or the epoch in which the CS was present. We measured each CS duration from the beginning of the first deflection of the extracellular potential to the time of the return to baseline potential (as indicated above panel Supplementary Fig. S1b). To reduce the bias in measurements, another author randomly verified the measurements and made independent measurements of randomly selected CS spikes, to crosscheck the results, while also being blind to the type of cell or the epoch in which the CS was present. Furthermore, random errors in measurements should not be prominent in a population study.

#### CS tuning to symbol and choice of hand

The CS responses in the symbol epoch and during movement were not selective for symbol or choice of hand respectively. To show this, we first calculated the contrast function (A – B) / (A + B) in the symbol epoch (50–250 ms after symbol onset) for preferences between the two symbols and in the movement epoch (50 ms before to 250 ms after the movement onset) for preferences between the hand movements and the symbols. To verify if this tuning were meaningful and not just due to extreme differences in sampling number and noise (due to sparseness in firing rate and low trial number), we generated a null distribution of spike times through a gamma distribution^45^ that was matched with the parameters of the experimental data (we obtained the shape parameter, $k$, the ISI distribution fit and took the scale parameter, $\theta$, as the inverse of firing rate) and calculated a similar tuning function on this null distribution. We found that the CS responses during the symbol (Supplementary Fig. S2a) or the movement epochs (Supplementary Fig. S2b) were not statistically different from a null distribution (symbol selectivity: *P* = 0.51; *t*-test; choice selectivity: *P* = 0.48; *t*-test).

### Statistics and reproducibility

All the experimental analyses were performed on CS from 25 P-cells, collected from two non-human primates.

### Reporting summary

Further information on research design is available in the Nature Research Reporting Summary linked to this article.

## Supplementary information

Supplementary Information

Reporting Summary

## Data Availability

All the relevant data that support the findings of this study are available at https://github.com/naveen-7/Cerebellum_reward. A reporting summary for this article is available as a Supplementary Information file. Source data are provided with this paper.

## Source data

Source Data

## Code availability

The codes used for the analyses that support the findings of this study are available from https://github.com/naveen-7/Cerebellum_reward.
