# Supplementary material for: Mid-lateral cerebellar complex spikes encode multiple independent reward-related signals during reinforcement learning
Source: Nat Commun. 2021 Nov 9;12:6475. doi: 10.1038/s41467-021-26338-0 (PMC8578621; doi:10.1038/s41467-021-26338-0)
Supplement: Supplementary file 1 — Supplementary Information [file 41467_2021_26338_MOESM1_ESM.pdf]

Mid-lateral cerebellar complex spikes encode multiple independent reward-related signals during reinforcement learning

Sendhilnathan et al.

Supplementary material

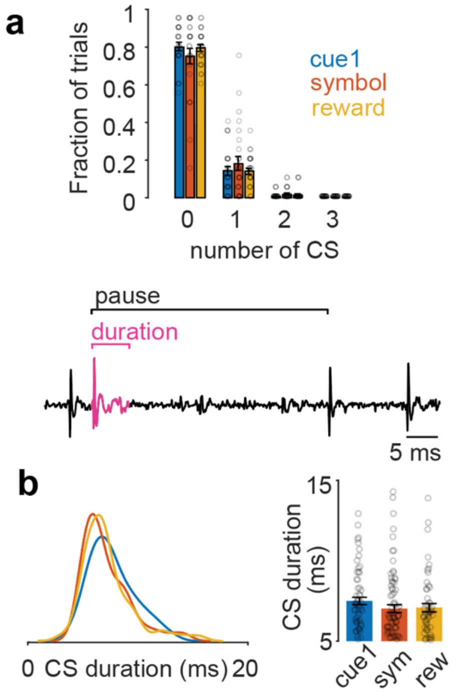

Supplementary Figure 1: CS responses properties in the OT condition

- **a.** Fraction of trials with 0,1,2 or 3 CS in the cue1 (blue), symbol (red) and reward (yellow) epochs. n = 25 cells.
- **b.** Duration of CS waveforms in the cue1 (blue) and symbol (red) and reward (yellow) epochs from n = 25 cells. Data shown as mean±s.e.m. Source data are provided as a Source Data file.

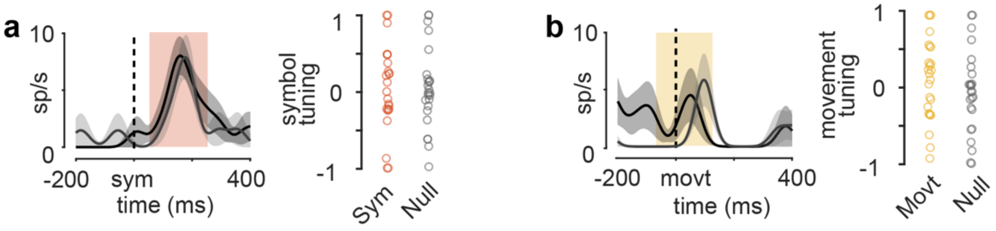

Supplementary Figure 2: CS responses were not tuned to sensorimotor parameters in the OT condition

- **a.** Left: CS responses from a representative P-cell for symbol 1 (gray) and symbol 2 (black). Shaded region shows the epoch where the CS activity was analyzed. Right: symbol tuning index for the P-cells (red) was not different from that of a null population (gray). See methods for more details. n = 25 cells.
- **b.** Left: CS responses from a different representative P-cell for left (gray) and right (black) hand release. Shaded region shows the epoch where the CS activity was analyzed. Right: movement tuning index for the P-cells (yellow) was not different from that of a null population (gray). See methods for more details. n = 25 cells. Data shown as mean±s.e.m. Source data are provided as a Source Data file.

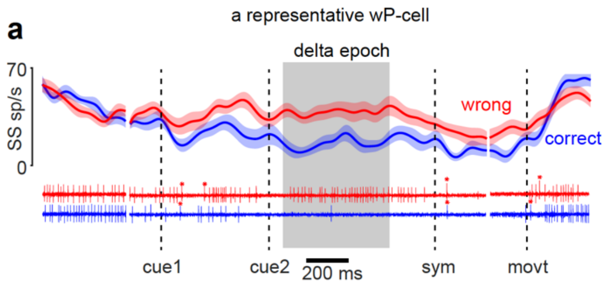

Supplementary Figure 3: SS and CS formed independent channels of neural encoding during reinforcement learning

- **a.** Top: A representative wP-cell SS activity during learning for correct (blue) and wrong (red) trials. Shaded region is the delta epoch (epoch with significant difference between recent correct and wrong trials). Bottom: Raw neural signals with SS and CS (marked by *) for one representative recent correct (blue) and wrong (red) trial during learning.
- **b.** Same as **Fig S3a**, but for a representative cP-cell. Data shown as mean±s.e.m.

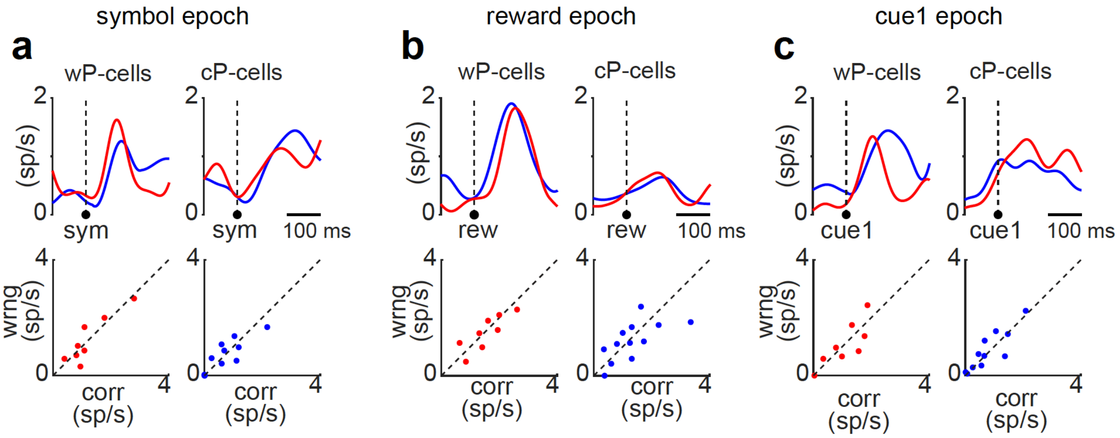

Supplementary Figure 4: CS activity does not carry information about the next trial outcome

- **a.** Top: CS activity during symbol epoch of L_{beg} separated into the next correct (blue) and wrong (red) trials for wP-cells (left, n = 11 wP-cells) and cP-cells (right, n = 14 cP-cells). Bottom: Scatter plot of peak neural activity during correct and wrong trials for individual wP-cells (left) and cP-cells (right). Same format as **Fig 2k**.
- **b.** Same as **a** but for activity in reward epoch.
- **c.** Same as **a** but for activity in cue 1 epoch.

Source data are provided as a Source Data file.

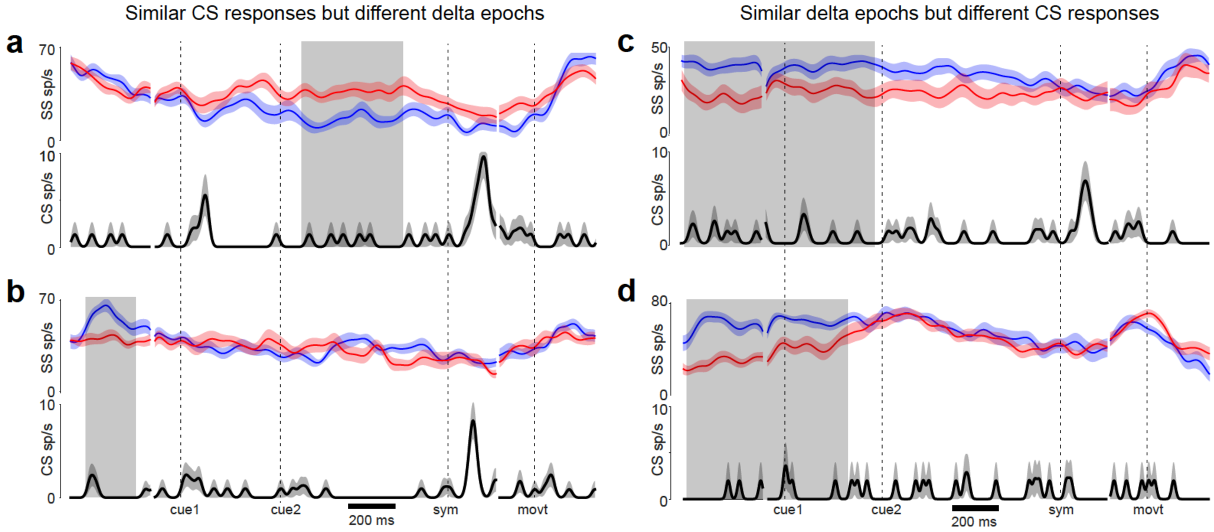

Supplementary Figure 5: Dissociation between SS delta epoch and CS responses
a and **b**: Two P-cells with similar CS responses (increased activity in symbol epoch) but very different SS delta epochs (gray shaded area).
c and **d**: Two P-cells with similar SS delta epochs (similar duration, both cP-cells) but with very different CS responses.
Data shown as mean±s.e.m.

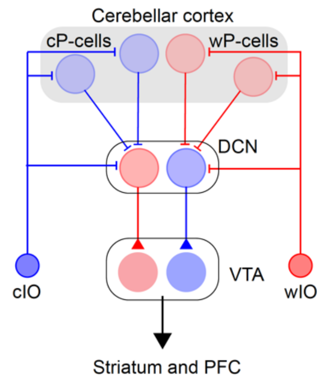

Supplementary Figure 6: A distributed circuit involving the cerebellum to drive reinforcement learning

cP-cells (blue) and wP-cells (red) in the cerebellar cortex process reward related information in their SS and collectively inhibit the deep cerebellar nucleus (DCN). Neurons from the inferior olive (IO) also project to the cerebellar cortex and DCN. The output from DCN is conveyed to the VTA neurons which then project to the striatum and the prefrontal cortex (PFC) for further reward processing.

Supplementary Table 1: CS Reponses

Response	# cells
C	12
S	11
R	18
C only	0
S only	1
R only	7
(C ∩ S) - R	3
(C ∩ R) - S	3
(R ∩ S) - C	2
C ∩ S	8
S ∩ R	7
C ∩ R	9
C ∩ S ∩ R	5
neither	3
