# Supplementary material for: Mid-lateral cerebellar complex spikes encode multiple independent reward-related signals during reinforcement learning
Source: Nat Commun. 2021 Nov 9;12:6475. doi: 10.1038/s41467-021-26338-0 (PMC8578621; doi:10.1038/s41467-021-26338-0)
Supplement: Supplementary file 2 — Reporting Summary [file 41467_2021_26338_MOESM2_ESM.pdf]

nature portfolio

Corresponding author(s): Naveen Sendhilnathan
Michael E. Goldberg

Last updated by author(s): Sep 28, 2021

Reporting Summary

Nature Portfolio wishes to improve the reproducibility of the work that we publish. This form provides structure for consistency and transparency in reporting. For further information on Nature Portfolio policies, see our Editorial Policies and the Editorial Policy Checklist.

Statistics

For all statistical analyses, confirm that the following items are present in the figure legend, table legend, main text, or Methods section.

n/a	Confirmed	
☐	☒	The exact sample size (*n*) for each experimental group/condition, given as a discrete number and unit of measurement
☐	☒	A statement on whether measurements were taken from distinct samples or whether the same sample was measured repeatedly
☐	☒	The statistical test(s) used AND whether they are one- or two-sided<br>*Only common tests should be described solely by name; describe more complex techniques in the Methods section.*
☒	☐	A description of all covariates tested
☐	☒	A description of any assumptions or corrections, such as tests of normality and adjustment for multiple comparisons
☐	☒	A full description of the statistical parameters including central tendency (e.g. means) or other basic estimates (e.g. regression coefficient) AND variation (e.g. standard deviation) or associated estimates of uncertainty (e.g. confidence intervals)
☐	☒	For null hypothesis testing, the test statistic (e.g. *F*, *t*, *r*) with confidence intervals, effect sizes, degrees of freedom and *P* value noted<br>*Give P values as exact values whenever suitable.*
☒	☐	For Bayesian analysis, information on the choice of priors and Markov chain Monte Carlo settings
☒	☐	For hierarchical and complex designs, identification of the appropriate level for tests and full reporting of outcomes
☐	☒	Estimates of effect sizes (e.g. Cohen's *d*, Pearson's *r*), indicating how they were calculated

Our web collection on statistics for biologists contains articles on many of the points above.

Software and code

Policy information about availability of computer code

Data collection	ePhys - glass-coated tungsten electrodes with an impedance of 0.8-1.2 MOhms (FHC), Hitachi microdrive, FHC Neurocraft head stage, Krohn-Hite filter, Spike 2- Micro 1401 system - CED electronics, NEI REX-VEX system. Video camera - Edmund Optics
Data analysis	MATLAB (Mathworks version 2016b), Spike2 CED electronics version 8, Track Mate-Image J

For manuscripts utilizing custom algorithms or software that are central to the research but not yet described in published literature, software must be made available to editors and reviewers. We strongly encourage code deposition in a community repository (e.g. GitHub). See the Nature Portfolio guidelines for submitting code & software for further information.

Data

Policy information about availability of data

All manuscripts must include a data availability statement. This statement should provide the following information, where applicable:

- Accession codes, unique identifiers, or web links for publicly available datasets
- A description of any restrictions on data availability
- For clinical datasets or third party data, please ensure that the statement adheres to our policy

All the relevant data are that support the findings of this study are available at https://github.com/naveen-7/Cerebellum_reward. A reporting summary for this article is available as a Supplementary Information file. Source data are provided with this paper.

Field-specific reporting

Please select the one below that is the best fit for your research. If you are not sure, read the appropriate sections before making your selection.

☒ Life sciences ☐ Behavioural & social sciences ☐ Ecological, evolutionary & environmental sciences

For a reference copy of the document with all sections, see nature.com/documents/nr-reporting-summary-flat.pdf

Life sciences study design

All studies must disclose on these points even when the disclosure is negative.

Sample size	Same size was not predetermined. We did not do a power analysis. We used the similar number of monkeys and cells that are consistent with other studies of single unit activities in awake behaving monkeys (Medina et al., 2008; Yang et al., 2014 etc).
Data exclusions	Only data with reliable complex spikes throughout the recordings were used. All other cells were excluded for the current data analysis
Replication	We performed the experiments and analyses on 2 monkeys. And the results between these monkeys were comparable. We also performed cross validation wherever applicable to ensure reproducibility
Randomization	Randomization was not performed because both the monkeys participated in all the experiments. The order of symbols appearing in the experiments was randomized so that the monkeys couldn't predict the upcoming symbol's identity.
Blinding	One of the authors manually made all these measurements while being blind to the type of cell or the epoch in which the CS was present. To reduce the bias in measurements, another author randomly verified the measurements and made independent measurements of randomly selected CS spikes, to crosscheck the results, while also being blind to the type of cell or the epoch in which the CS was present. The investigators were blinded to group allocation during data analysis. Furthermore, random errors in measurements should not be prominent in a population study.

Reporting for specific materials, systems and methods

We require information from authors about some types of materials, experimental systems and methods used in many studies. Here, indicate whether each material, system or method listed is relevant to your study. If you are not sure if a list item applies to your research, read the appropriate section before selecting a response.

Materials & experimental systems

n/a	Involved in the study	
☒	☐	Antibodies
☒	☐	Eukaryotic cell lines
☒	☐	Palaeontology and archaeology
☐	☒	Animals and other organisms
☒	☐	Human research participants
☒	☐	Clinical data
☒	☐	Dual use research of concern

Methods

n/a	Involved in the study	
☒	☐	ChIP-seq
☒	☐	Flow cytometry
☒	☐	MRI-based neuroimaging

Animals and other organisms

Policy information about studies involving animals; ARRIVE guidelines recommended for reporting animal research

Laboratory animals	We performed all experiments on two adult male monkeys (Macaca mulatta), B (age 17 years) and S (age 7 years), each weighing 10-11 kg each, for the experiments.
Wild animals	The study did not involve wild animals
Field-collected samples	No field collected samples were used in the study.
Ethics oversight	All experimental protocols were approved by the Animal Care and Use Committees at Columbia University and the New York State Psychiatric Institute, and complied with the guidelines established by the Public Health Service Guide for the Care and Use of Laboratory Animals.

Note that full information on the approval of the study protocol must also be provided in the manuscript.
